# Ral GTPase promotes metastasis of pancreatic ductal adenocarcinoma *via* elevation of TGF-β1 production

**DOI:** 10.1016/j.jbc.2023.104754

**Published:** 2023-04-26

**Authors:** Mingxin Cao, Xinming Li, Duc-Anh Trinh, Shingo Yoshimachi, Kota Goto, Natsumi Sakata, Masaharu Ishida, Hideo Ohtsuka, Michiaki Unno, Yuxia Wang, Ryutaro Shirakawa, Hisanori Horiuchi

**Affiliations:** 1 Department of Molecular and Cellular Biology, Institute of Development, Aging and Cancer, Tohoku University, Sendai, Miyagi, Japan; 2 Department of Oral Cancer Therapeutics, Graduate School of Dentistry, Tohoku University, Sendai, Miyagi, Japan; 3 State Key Laboratory of Oral Diseases & National Clinical Research Center for Oral Diseases & Department of Oral and Maxillofacial Surgery, West China Hospital of Stomatology, Sichuan University, Chengdu, China; 4 School and Hospital of Stomatology, Tianjin Medical University, Tianjin, China; 5 Tianjin Key Laboratory of Oral and Maxillofacial Function Reconstruction, Tianjin Stomatological Hospital, The Affiliated Stomatological Hospital of Nankai University, Tianjin, China; 6 Department of Surgery, Graduate School of Medicine, Tohoku University, Sendai, Miyagi, Japan

**Keywords:** metastasis, pancreatic ductal adenocarcinoma, RalGAP, Ral GTPase, TGF-β1

## Abstract

Pancreatic ductal adenocarcinoma (PDAC), caused by activating mutations in K-Ras, is an aggressive malignancy due to its early invasion and metastasis. Ral GTPases are activated downstream of Ras and play a crucial role in the development and progression of PDAC. However, the underlying mechanisms remain unclear. In this study, we investigated the mechanism of Ral-induced invasion and metastasis of PDAC cells using RalGAPβ-deficient PDAC cells with highly activated Ral GTPases. Array analysis and ELISA revealed increased expression and secretion of TGF-β1 in RalGAPβ-deficient PDAC cells compared to control cells. Blockade of TGF-β1 signaling suppressed RalGAPβ deficiency-enhanced migration and invasion *in vitro* and metastasis *in vivo* to levels similar to controls. Phosphorylation of c-Jun N-terminal kinase, a repressor of TGF-β1 expression, was decreased by RalGAPβ deficiency. These results indicate that Ral contributes to invasion and metastasis of PDAC cells by elevating autocrine TGF-β1 signaling at least in part by decreasing c-Jun N-terminal kinase activity.

Pancreatic ductal adenocarcinoma (PDAC) is an aggressive malignancy with a 5-year survival rate of approximately 10% (1). Early invasion and metastasis are the main reasons for this poor survival rate (2). The driver gene of most PDAC cells is known to be K-Ras with an active mutation (2). K-Ras is a small GTPase that mediates cell proliferation by acting as a switch (3). It is normally present in an inactive GDP-bound form in resting cells, and upon activation of growth factor receptors, GDP-K-Ras is converted to an active GTP-bound form *via* the GDP/GTP exchange reaction mediated by its GDP/GTP exchange factors (GEFs). GTP-K-Ras transduces proliferation signals through downstream effectors until it is inactivated by GTPase activating proteins (GAPs). Because mutant K-Ras is resistant to GAPs, it remains fixed in the GTP-bound active form and continuously transmits “on” signals for cell proliferation (4).

Ral GTPases, including RalA and RalB, are members of Ras family GTPases. Like other GTPases, Ral is activated by RalGEFs and inactivated by RalGAPs (5, 6). To date, seven RalGEFs have been identified, named RalGDS, RGL1, RGL2/Rlf, RGL3, Rgr/RGL4, RalGPS1, and RalGPS2. Since RalGDS, RGL1, RGL2/Rlf, and RGL3 are direct effectors of active Ras, Ral is activated downstream of Ras (7). RalGAPs are heterodimers consisting of a catalytic α1 or α2 subunit and a common β subunit, forming two distinct complexes, RalGAP1 (α1-β) and RalGAP2 (α2-β) (6, 8). Most tissues express both α subunits, with α1 predominant in the brain and α2 in the liver and lung (8). In the pancreas, the expression levels of α1 and α2 subunits are similar (9).

Ral plays a critical role in the oncogenic Ras-induced transformation of human cells (10, 11). Activation of Ral is important in chemically induced skin carcinogenesis (12). Furthermore, a forward genetic screen in mice identified RalGAPα2 as a major suppressor of hepatocellular carcinoma (13). We have previously shown that Ral exerts crucial effects on the development and progression of several cancers, including bladder cancer, oral cancer, prostate cancer, and colorectal cancer (14, 15, 16, 17). In pancreatic cancer, Ral has been shown to be highly activated in cancer tissues with K-Ras mutations (18). We have previously generated RalGAPβ-deficient PDAC cells using two cell lines, MIA PaCa-2 and PANC-1, and found that these cells have high Ral activity and exhibit high invasive and metastatic capacities (9). However, since Ral can interact with various downstream effectors such as the exocyst complex, RalBP1, phospholipase D, and M-Sec, it remains unclear how Ral regulates invasion and metastasis of PDAC cells. In the present study, we show that activation of Ral increases transforming growth factor beta 1 (TGF-β1) production in PDAC cells, which promotes their invasion and metastasis.

## Results

### RalGAPβ deficiency enhances TGF-β1 expression in PDAC cells

We have previously shown that RalGAPβ-deficient PDAC cells have high activities of RalA and RalB and exhibit increased migration and invasion (9). To identify the factors underlying these behaviors, we used two clonal MIA PaCa-2 KO cell lines (KO1 and KO2) previously generated by CRISPR-Cas9 genome editing (9) and compared each with their parental (WT) or control (Con) cells. "Con" refers to a clonal MIA PaCa-2 cell line obtained by the same CRISPR-Cas9 treatment but without indel mutations in the RalGAPβ genomic region. These cell lines were compared in four sets as follows: KO1 *versus* WT, KO1 *versus* Con, KO2 *versus* WT, and KO2 *versus* Con. We listed genes with more than 2-fold changes in each comparison (Table S1, Supporting information). We then narrowed down the candidates by selecting genes that were commonly listed in the four comparisons (Tables S2 and S3).

**Figure 1:**
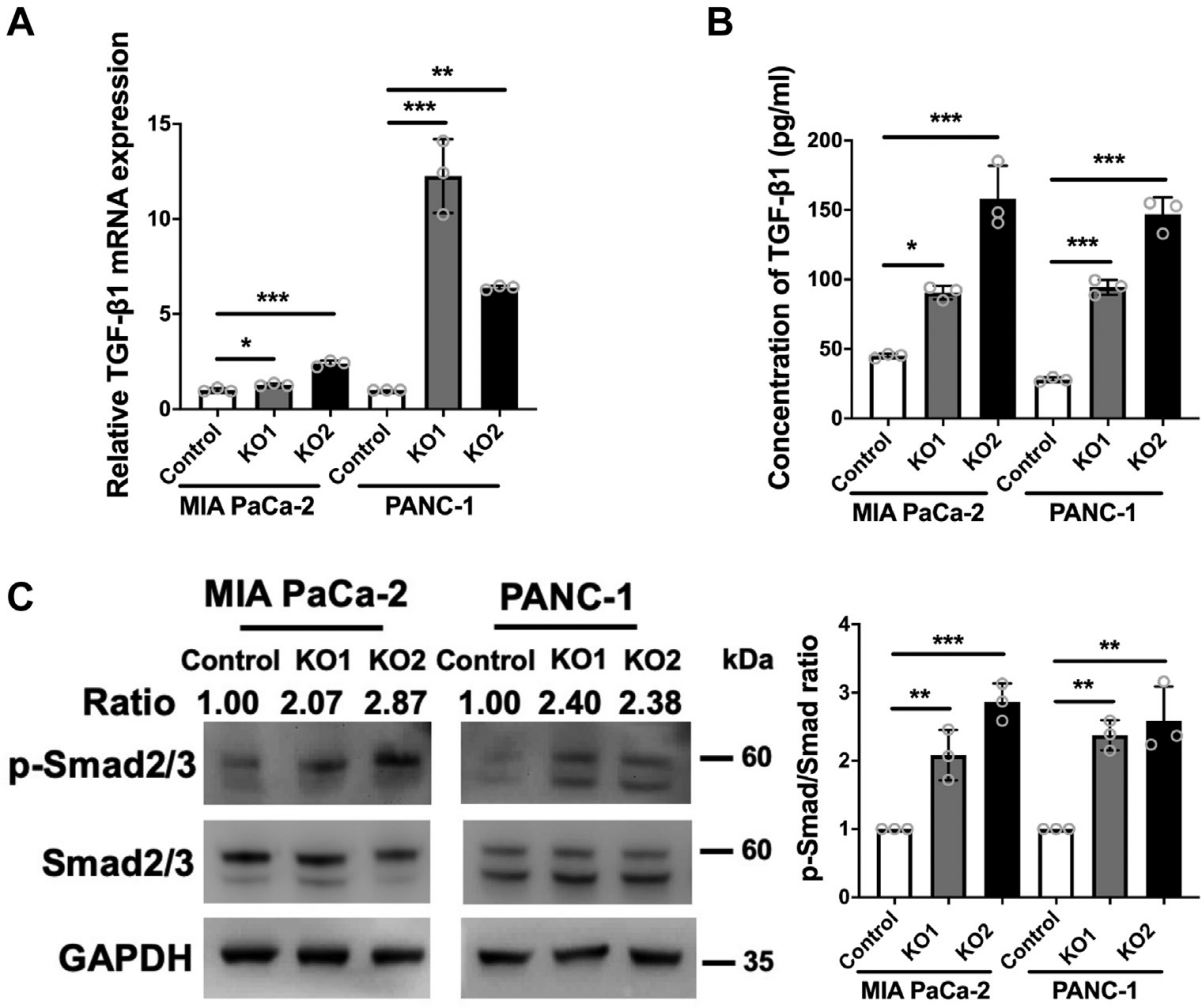
**RalGAPβ deficiency promotes TGF-β1 expression and Smad2/3 phosphorylation in PDAC cells**. *A* and *B*, TGF-β1 mRNA levels (*A*) and protein levels in the culture media (*B*) were quantified in RalGAPβ-deficient and control MIA PaCa-2 and PANC-1 cells by qPCR and ELISA. *C*, phosphorylated Smad2/3 (p-Smad2/3), total Smad2/3, and GAPDH were evaluated in RalGAPβ-deficient and control MIA PaCa-2 and PANC-1 cells by Western blot. The ratios of p-Smad2/3 to Smad2/3 were quantified. Data are means ± SD of three independent experiments. ∗*P* ＜ 0.05; ∗∗*P* ＜ 0.01; ∗∗∗*P* ＜ 0.001. GAP, GTPase activating protein; PDAC, pancreatic ductal adenocarcinoma; qPCR, quantitative real-time PCR; TGF-β1, transforming growth factor beta 1.

Eight genes were consistently upregulated in RalGAPβ-KO1 and RalGAPβ-KO2 MIA PaCa-2 cells (fold change ≥ 2.0, *P* ＜ 0.05), whereas 13 mRNAs were downregulated compared with parental or control MIA PaCa-2 cells (fold change ≤ −2.0, *P* ＜ 0.05). Of these, we found that TGF-β1 was greatly and consistently increased in RalGAPβ-deficient cells. In KO1 cells, TGF-β1 expression was 2.55- and 2.66-fold higher than in parental and control cells, respectively. KO2 cells also showed higher TGF-β1 expression than parental and control cells (4.12- and 5.69-fold, respectively). Since TGF-β1 has been implicated in cell motility and cancer progression (19), and it has been reported that cancer cells from late-stage of PDAC secrete large amounts of TGF-β1 (20), we here focused on TGF-β1 for further analysis. Quantitative real-time PCR (qPCR) analysis revealed increased expression of TGF-β1 in RalGAPβ-deficient MIA PaCa-2 cells (Fig. 1*A*). Furthermore, RalGAPβ-deficient PANC-1 cells showed a greater increase in TGF-β1 mRNA levels than control cells (Fig. 1*A*). We observed no consistent changes in other members of the TGF-β family, including TGF-β2, TGF-β3, and inhibin βA (Fig. S1*A*). ELISA assays showed that TGF-β1 protein was significantly increased in the culture media of RalGAPβ-deficient MIA PaCa-2 and PANC-1 cells relative to controls (Fig. 1*B*). These results indicate that TGF-β1 production was upregulated in both RalGAPβ-deficient MIA PaCa-2 and PANC-1 cells.

Members of the TGF-β family typically exert their function in an autocrine manner through receptor activation and expression of target genes, such as TMEPAI and PAI-1. Specifically, TGF-β binds and activates TGF-β type II receptor serine/threonine kinases (TβRII), which in turn phosphorylate and activate type I receptors (TβRI). The activated TβRI kinases then phosphorylate downstream mediators such as Smad2 and Smad3 to initiate the TGF-β signaling pathway (21). Among the target genes of TGF-β, we found no consistent change in the mRNA expression levels of TMEPAI in RalGAPβ-deficient cells, but a clear increase was observed for PAI-1 (Fig. S1*B*). As a more direct measure of TGF-β receptor activation, we observed increased Smad2/3 phosphorylation in RalGAPβ-deficient PDAC cells (Fig. 1*C*), suggesting that TGF-β1 signaling is activated by RalGAPβ deficiency.

### Suppression of TGF-β1 expression attenuates RalGAPβ deficiency-enhanced migration and invasion

**Figure 2:**
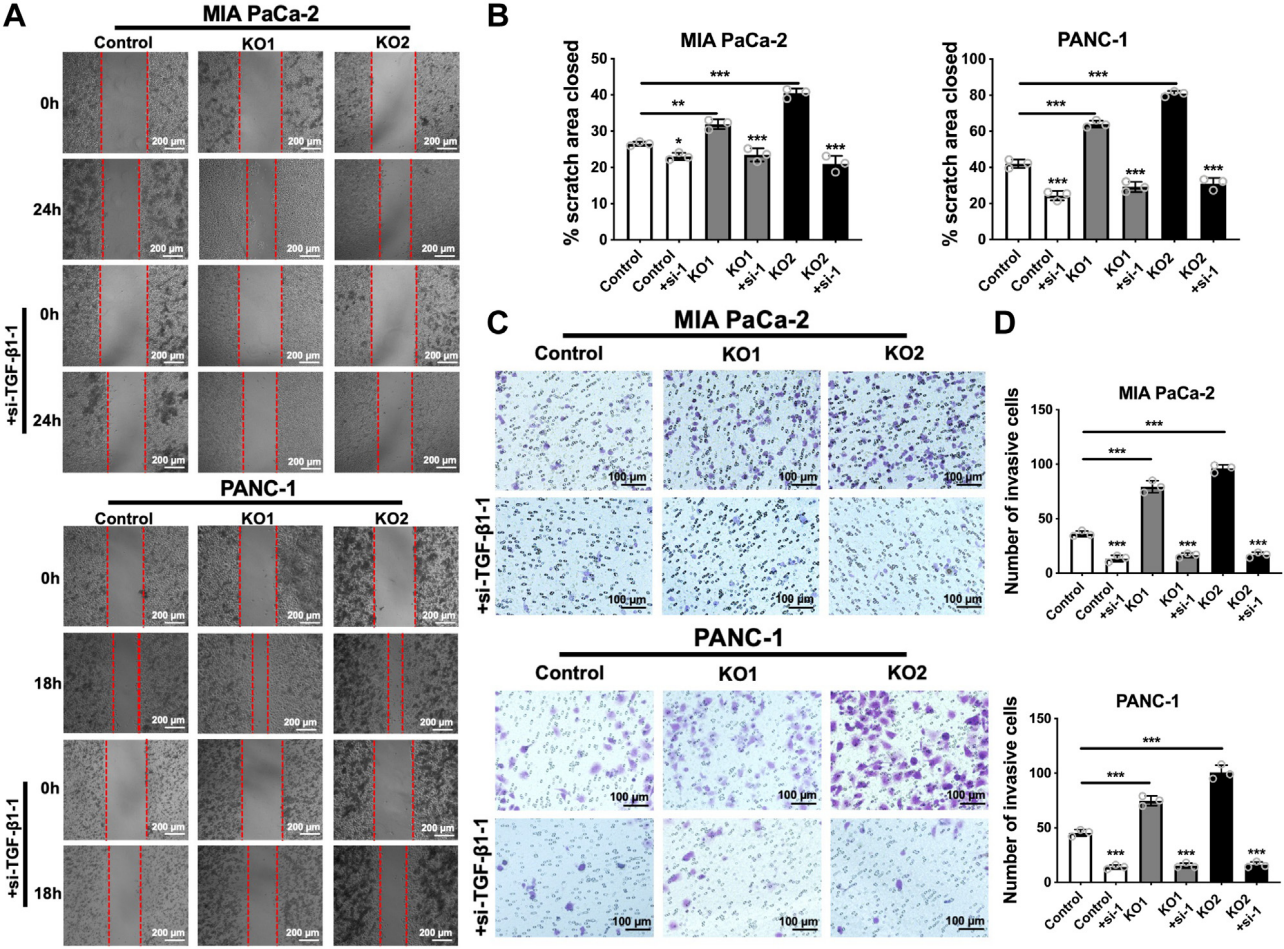
**Suppression of TGF-β1 expression attenuates RalGAPβ deficiency-enhanced migration and invasion of PDAC cells.***A*, wound healing assay of RalGAPβ-deficient and control MIA PaCa-2 and PANC-1 cells with or without TGF-β1-siRNA knockdown. *B*, quantification of the closed scratch area (%) of the wound healing assay. *C*, transwell invasion assay of RalGAPβ-deficient and control MIA PaCa-2 and PANC-1 cells with or without TGF-β1-siRNA knockdown. *D*, number of invasive cells in the transwell invasion assay. Comparisons within each cell line were conducted between control and siRNA-treated groups. Data are means ± SD of three independent experiments. ∗*P* ＜ 0.05; ∗∗*P* ＜ 0.01; ∗∗∗*P* ＜ 0.001.GAP, GTPase activating protein; PDAC, pancreatic ductal adenocarcinoma; TGF-β1, transforming growth factor beta 1.

To investigate whether increased TGF-β1 production mediates RalGAPβ deficiency-enhanced migration and invasion of PDAC cells, we used siRNAs to knock down TGF-β1 expression in MIA PaCa-2 and PANC-1 cells. Knockdown efficiency was validated by qPCR (Fig. S1, *C*–*E*). In wound healing and transwell invasion assays, RalGAPβ-deficient PDAC cells showed a significant increase in both migratory area and invasive capacity, consistent with our previous data (9). Suppression of TGF-β1 expression in RalGAPβ-KO1 and RalGAPβ-KO2 MIA PaCa-2 and PANC-1 cells almost abolished their enhanced migration and invasion (Figs. 2, S2 and S3). These results indicate that TGF-β1 production is required for the enhanced migration and invasion of RalGAPβ KO PDAC cells.

### Blocking autocrine TGF-β1 signaling abrogates RalGAPβ deficiency-enhanced migration and invasion

**Figure 3:**
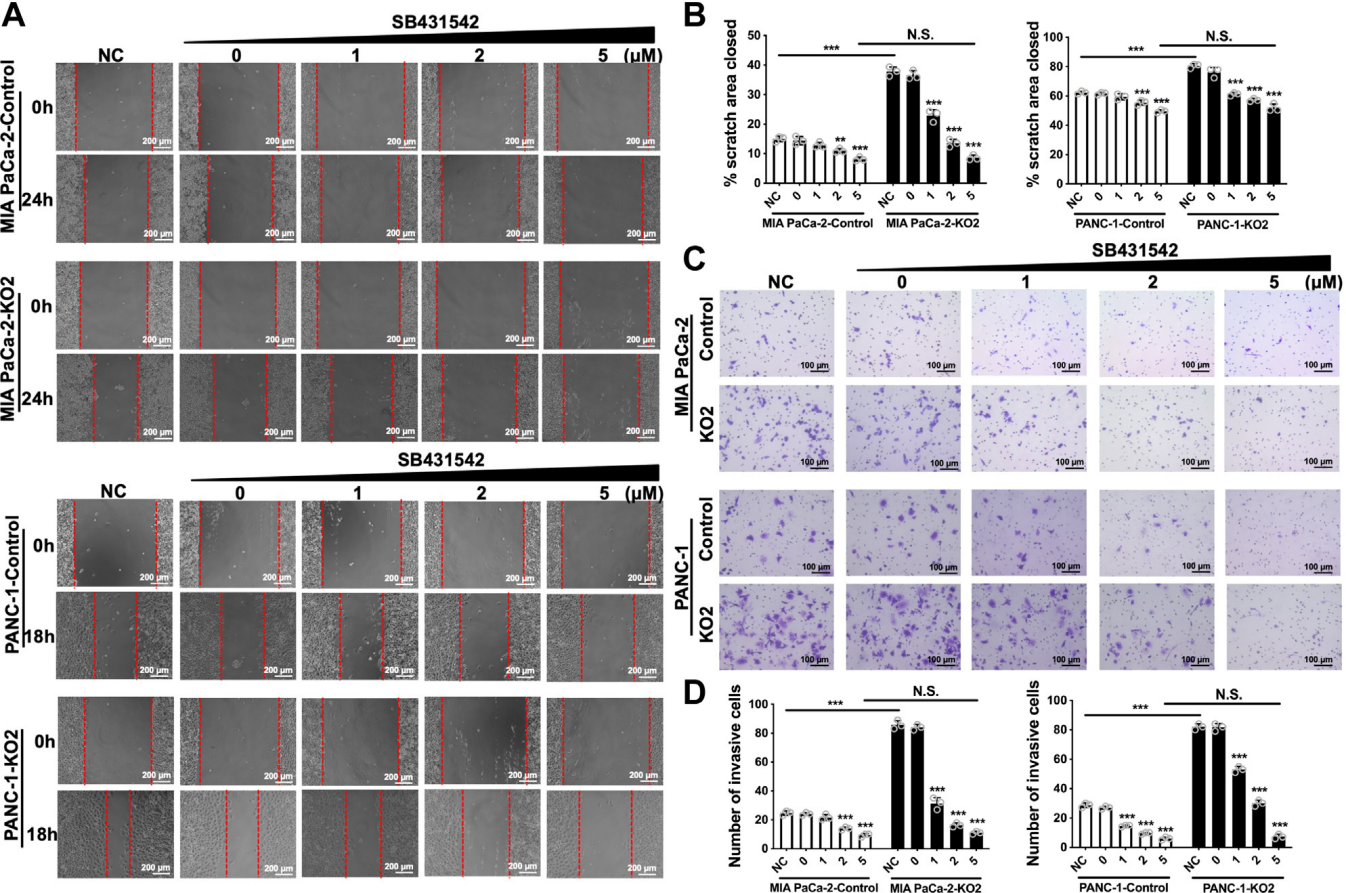
**Blockade of autocrine TGF-β1 signaling abrogates RalGAPβ deficiency-enhanced migration and invasion of PDAC cells.***A*, wound healing assay of RalGAPβ-deficient and control MIA PaCa-2 and PANC-1 cells without (NC) or with different concentrations of the TGF-β type I receptor inhibitor SB431542. NC is the negative control without treatment, and 0 μM is the DMSO control. *B*, quantification of the closed scratch area (%) of the wound healing assay. *C*, transwell invasion assay of RalGAPβ-deficient and control MIA PaCa-2 and PANC-1 cells without or with the TGF-β receptor inhibitor SB431542. *D*, number of invasive cells of the transwell invasion assay. Comparisons within each cell line were conducted with reference to the NC group. Data are means ± SD of three independent experiments. N.S., not significant; ∗∗*P* ＜ 0.01; ∗∗∗*P* ＜ 0.001. DMSO, dimethyl sulfoxide; GAP, GTPase activating protein; PDAC, pancreatic ductal adenocarcinoma; TGF-β1, transforming growth factor beta 1.

To investigate whether TGF-β1 acts in an autocrine manner to promote PDAC cell migration and invasion, we used the small molecule TβRI kinase inhibitor SB431542 to block the TGF-β signaling pathway (22). As shown in Figure 3, *A* and *B*, SB431542 reduced RalGAPβ deficiency-enhanced migration in a concentration-dependent manner. At a concentration of 5 μM, SB431542 almost completely abolished the effect of RalGAPβ deficiency in MIA PaCa-2 and PANC-1 cells. Similarly, the transwell invasion assay showed that SB431542 concentration dependently decreased the invasive activity enhanced by RalGAPβ deficiency and almost completely abrogated the effect at a concentration of 5 μM (Fig. 3, *C* and *D*). These data suggest that RalGAPβ deficiency promotes PDAC cell migration and invasion through increased TGF-β1 expression and autocrine TGF-β1 signaling *in vitro*.

### RalGAP-mediated inhibition of Ral activity attenuates TGF-β1 expression, migration, and invasion

**Figure 4:**
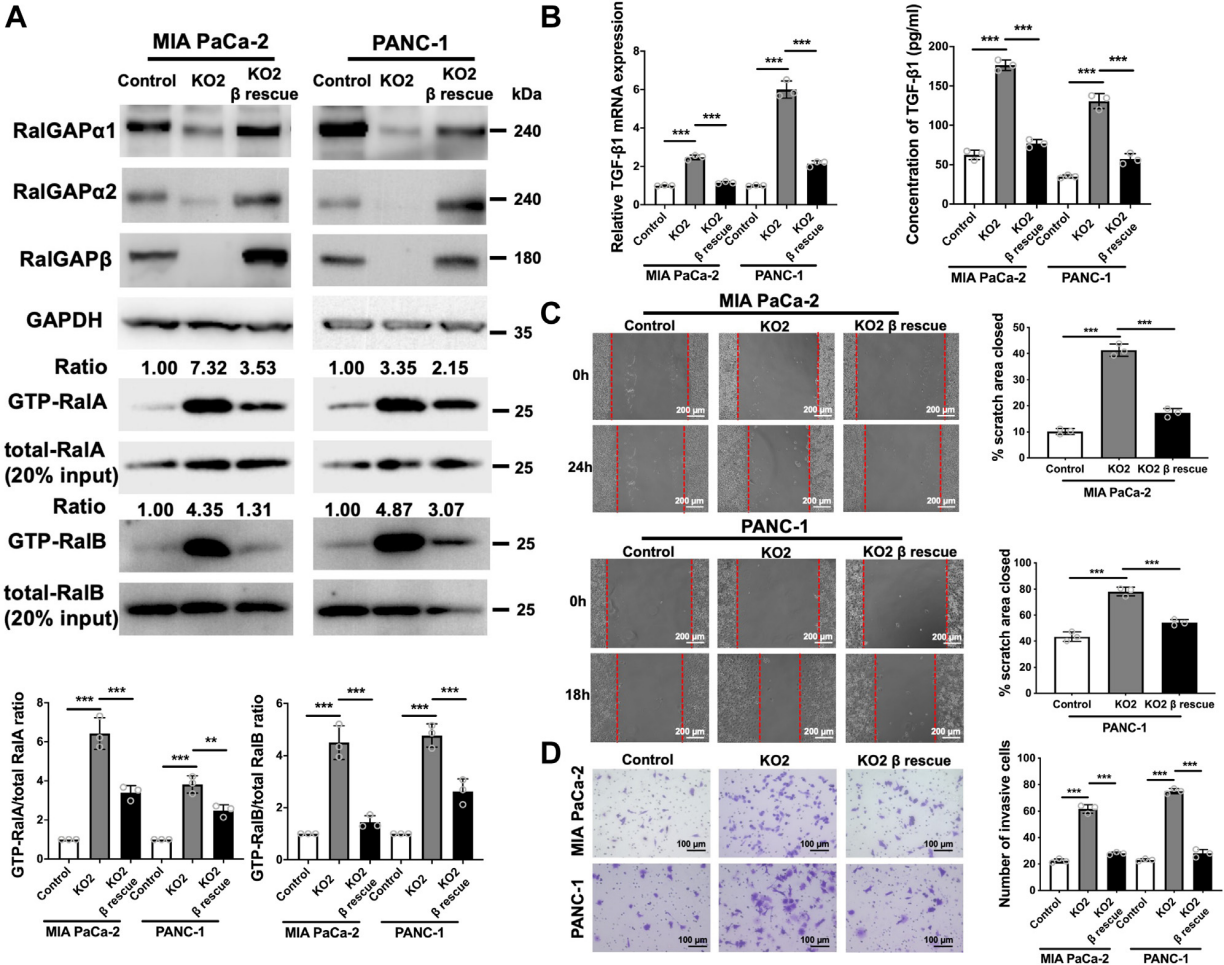
**Rescuing RalGAPβ expression inhibits Ral activation, migration, invasion, and TGF-β1 expression in PDAC cells.***A*, GTP-bound RalA and RalB were assessed by GST-Sec5 pull-down assays in control, RalGAPβ-deficient, and RalGAPβ-rescued MIA PaCa-2 and PANC-1 cells. RalGAPα1, RalGAPα2, RalGAPβ, GAPDH, and total RalA and RalB were analyzed by Western blot. The ratios of GTP-bound RalA/B to total RalA/B were quantified. *B*, TGF-β1 mRNA and protein in the culture media were examined in control, RalGAPβ-deficient, and RalGAPβ-rescued MIA PaCa-2 and PANC-1 cells by qPCR and ELISA. *C* and *D*, wound healing assay (*C*) and transwell invasion assay (*D*) of control, RalGAPβ-deficient, and RalGAPβ-rescued MIA PaCa-2 and PANC-1 cells. Representative images are shown on the *left* and quantitative results on the *right*. Data are means ± SD of three independent experiments. ∗∗*P* ＜ 0.01; ∗∗∗*P* ＜ 0.001. GAP, GTPase activating protein; PDAC, pancreatic ductal adenocarcinoma; TGF-β1, transforming growth factor beta 1.

To investigate whether RalGAPβ deficiency-increased TGF-β1 expression, migration, and invasion were directly due to Ral activation, we rescued RalGAPβ expression in RalGAPβ-deficient MIA PaCa-2 and PANC-1 cells. Western blot demonstrated that RalGAPβ expression was recovered in RalGAPβ-rescued cells to levels comparable to or higher than those in control cells (Figs. 4*A* and S4*A*). RalGAPα1 and RalGAPα2 were also increased in RalGAPβ-rescued cells, consistent with our previous observation that RalGAPβ is necessary for the protein stability of RalGAPα1 and RalGAPα2 (8). Pull-down assays showed that the activities of RalA and RalB were inhibited by RalGAPβ reexpression (Figs. 4*A* and S4*A*). Importantly, we found decreased transcription and secretion of TGF-β1 in RalGAPβ-rescued MIA PaCa-2 and PANC-1 cells compared to RalGAPβ-deficient cells (Figs. 4*B* and S4*B*). Migration and invasion were also suppressed by RalGAPβ reexpression as assessed by wound healing and transwell invasion assays (Figs. 4, *C* and *D* and S4, *C* and *D*).

**Figure 5:**
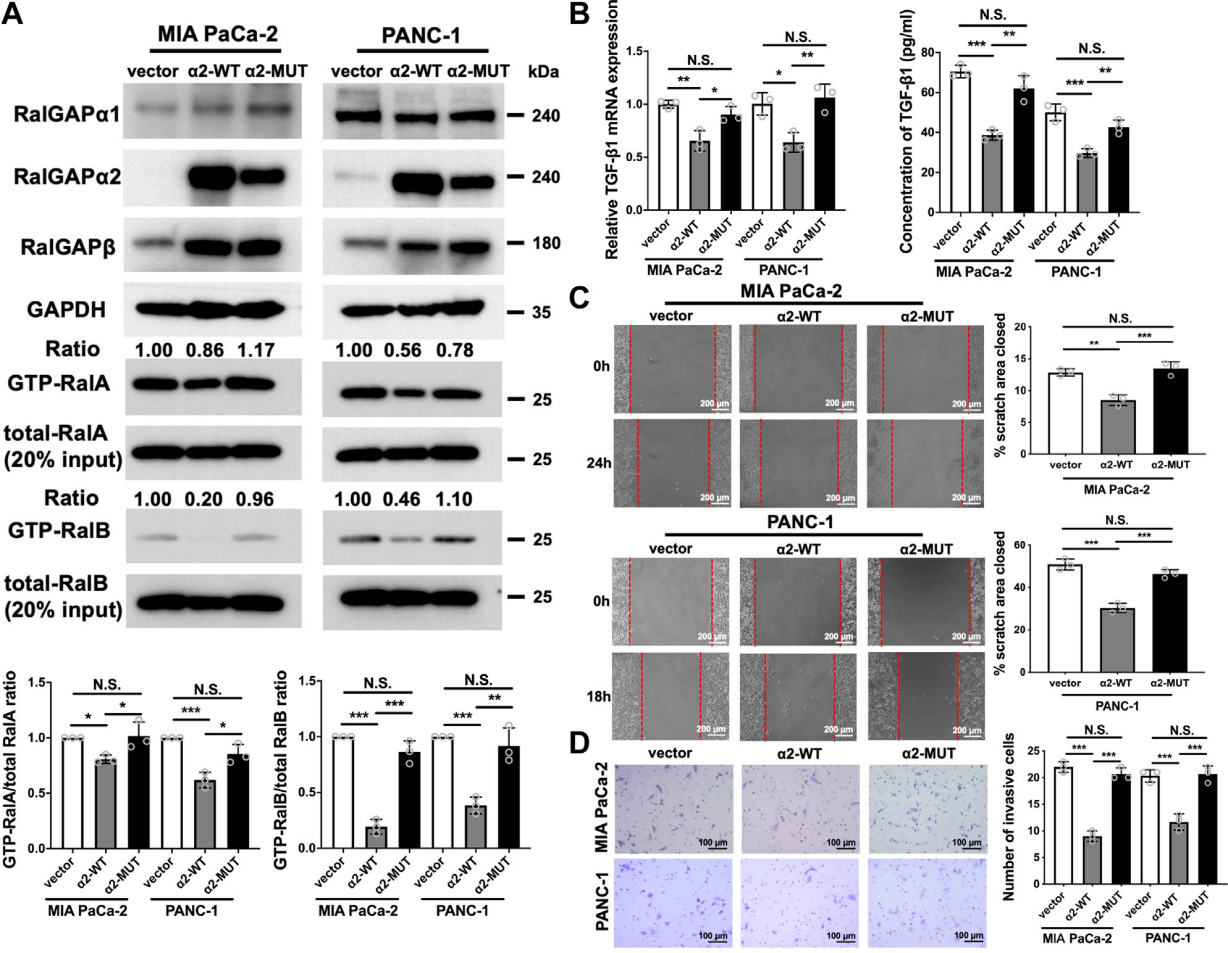
**Overexpression of RalGAPα2 inhibits Ral activation, migration, invasion, and TGF-β1 expression in PDAC cells.***A*, GTP-bound RalA and RalB were assessed by GST-Sec5 pull-down assays in MIA PaCa-2 and PANC-1 cells stably expressing vector alone, RalGAPα2-WT, or RalGAPα2-MUT (N1742K mutant lacking RalGAP activity). RalGAPα1, RalGAPα2, RalGAPβ, GAPDH, and total RalA and RalB were assessed by Western blot. The ratios of GTP-bound RalA/B to total RalA/B were quantified. *B*, TGF-β1 mRNA and protein in the culture media were examined in MIA PaCa-2 and PANC-1 cells stably expressing vector alone, RalGAPα2-WT, or RalGAPα2-MUT by qPCR and ELISA. *C* and *D*, wound healing assay (*C*) and transwell invasion assay (*D*) of vector alone, RalGAPα2-WT, and RalGAPα2-MUT MIA PaCa-2 and PANC-1 cells. Representative images are shown on the *left* and quantitative results on the *right*. Data are means ± SD of three independent experiments. N.S., not significant; ∗*P* ＜ 0.05; ∗∗*P* ＜ 0.01; ∗∗∗*P* ＜ 0.001. GAP, GTPase activating protein; GST, glutathione-S-transferase; PDAC, pancreatic ductal adenocarcinoma; qPCR, quantitative real-time PCR; TGF-β1, transforming growth factor beta 1.

We next examined the effect of Ral inactivation on TGF-β1 production in PDAC cells. Overexpression of RalGAPα2 in MIA PaCa-2 and PANC-1 parental cells, both of which have low levels of endogenous RalGAPα2 expression, decreased the GTP-bound active form of RalA and RalB as assessed by the Sec5 pull-down assay. The RalGAPα2 N1742K mutant (RalGAPα2-MUT), which lacks RalGAP activity, had no effect on the GTP-bound Ral levels (Fig. 5*A*). MIA PaCa-2 and PANC-1 cells expressing RalGAPα2-WT, but not RalGAPα2-MUT, showed decreased transcription and secretion of TGF-β1 (Fig. 5*B*). This was also the case for migration and invasion capacities (Fig. 5, *C* and *D*). These results suggest that activation of Ral promotes migration and invasion through increased production of TGF-β1.

### RalGAPβ deficiency promotes TGF-β1 expression *via* downregulation of JNK phosphorylation

**Figure 6:**
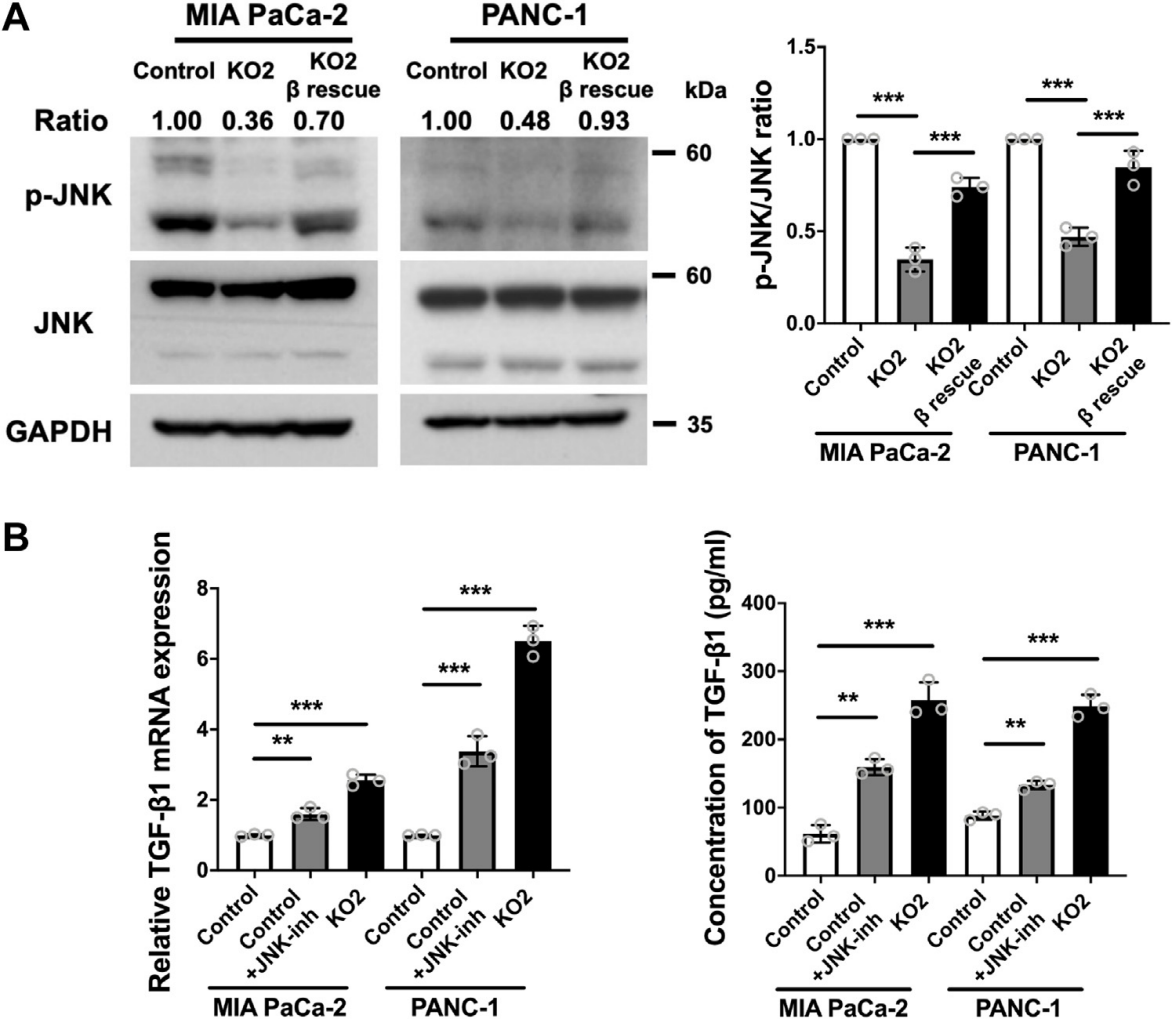
**RalGAPβ deficiency promotes TGF-β1 expression via downregulation of JNK phosphorylation.***A*, protein levels of phosphorylated JNK (p-JNK), total JNK, and GAPDH in control, RalGAPβ-deficient, and RalGAPβ-rescued MIA PaCa-2 and PANC-1 cells were determined by Western blot. The ratios of p-JNK to JNK were quantified. *B*, TGF-β1 mRNA and protein in the culture media were examined in control MIA PaCa-2 and PANC-1 cells without or with the JNK inhibitor SP600125 (10 μM) and RalGAPβ-deficient MIA PaCa-2 and PANC-1 cells by qPCR and ELISA. Data are means ± SD of three independent experiments. ∗∗*P* ＜ 0.01; ∗∗∗*P* ＜ 0.001. GAP, GTPase activating protein; JNK, c-Jun N-terminal kinase; TGF-β1, transforming growth factor beta 1.

We then focused on what downstream signals of Ral contribute to increased TGF-β1 transcription. It has been reported that c-Jun N-terminal kinase (JNK) deficiency promotes TGF-β1 transcription by decreasing c-Jun binding to the negative regulatory region of the TGF-β1 promoter (23). Thus, we examined whether the JNK pathway mediates the Ral-induced TGF-β1 transcription observed here. In RalGAPβ-deficient MIA PaCa-2 and PANC-1 cells, JNK phosphorylation was significantly decreased compared to both control and RalGAPβ-rescued cells (Fig. 6*A*). We then used the JNK protein kinase inhibitor SP600125 to test the effect of blocking the JNK pathway on RalGAPβ deficiency-induced TGF-β1 expression. This showed that the transcription and secretion of TGF-β1 were increased to some extent after treatment with the JNK inhibitor (Fig. 6*B*). Thus, the effects of RalGAPβ deficiency on increased TGF-β1 expression were mediated, at least in part, by downregulation of JNK phosphorylation.

### Autocrine TGF-β1 signaling is essential for RalGAPβ deficiency-induced metastasis *in vivo*

**Figure 7:**
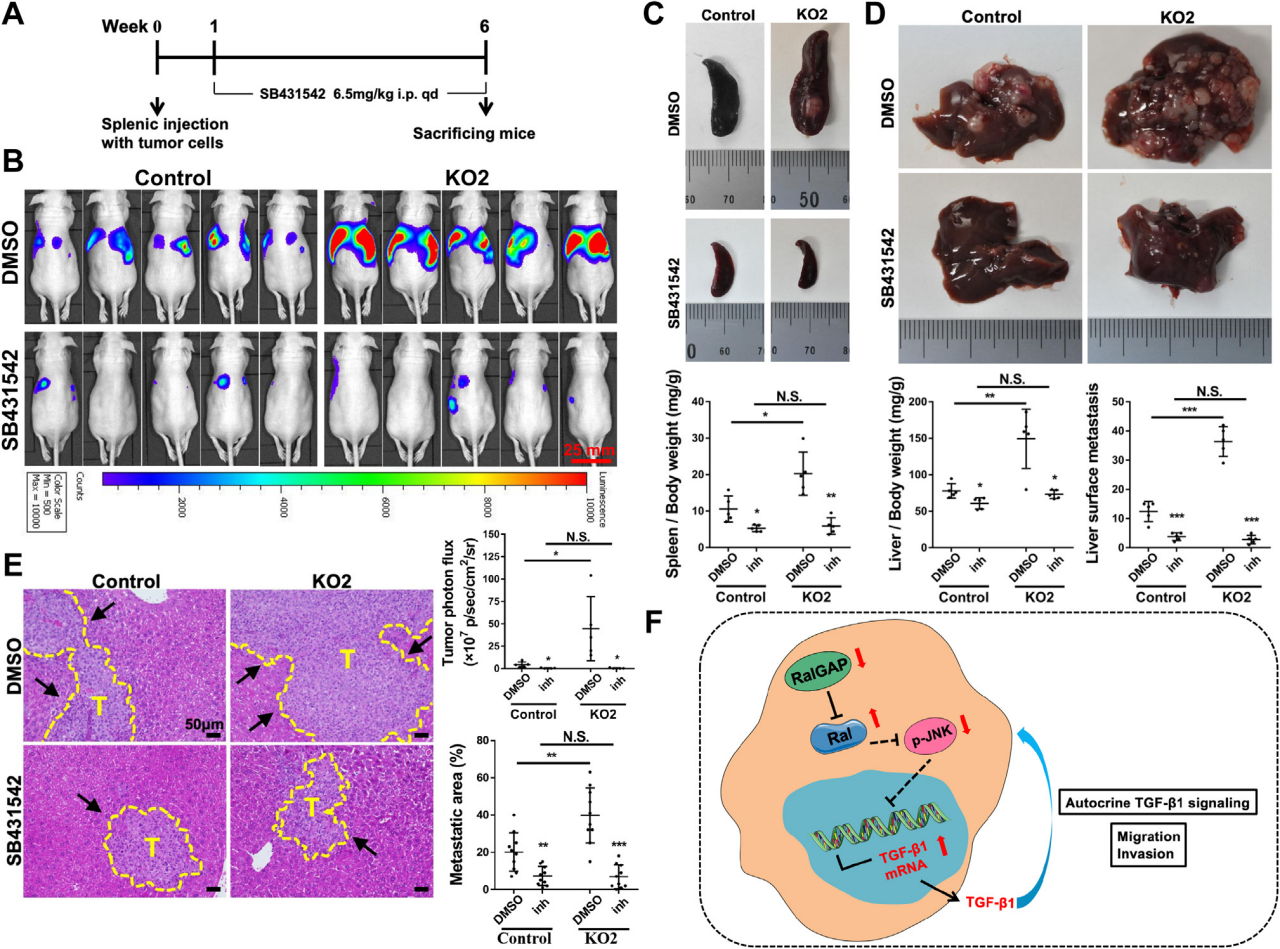
**Blockade of TGF-β1 signaling abrogates RalGAPβ deficiency-induced metastasis *in vivo*.***A*, schema representing the animal experiment procedures. *B-E*, RalGAPβ-deficient or control MIA PaCa-2-luciferase cells were injected into the spleens of nude mice. Luminescence signals by *in vivo* imaging (*B*), spleen weight (*C*), liver weight and liver surface metastasis (*D*), and liver metastasis by histopathological analysis (*E*) were evaluated in mice treated with or without SB431542. Representative images and corresponding quantitative results are shown. *F*, proposed model illustrating the role of RalGAP-Ral-JNK-TGF-β1 in PDAC progression. The scale bar represents 50 μm. Comparisons within each cell lines were conducted between inhibitor and DMSO group. Data are represented as means ± SD. T, tumor; N.S., not significant; ∗*P* ＜ 0.05; ∗∗*P* ＜ 0.01; ∗∗∗*P* ＜ 0.001. DMSO, dimethyl sulfoxide; GAP, GTPase activating protein; JNK, c-Jun N-terminal kinase; qPCR, quantitative real-time PCR; PDAC, pancreatic ductal adenocarcinoma; TGF-β1, transforming growth factor beta 1.

We have previously shown that RalGAP-deficient MIA PaCa-2 cells injected into the spleen of mice exhibit increased local proliferation and liver metastasis compared to control cells (9). Using this splenic injection model, we investigated the contribution of TGF-β1 to Ral-enhanced metastasis *in vivo*. One week after injection of RalGAPβ-deficient or control MIA PaCa-2 cells stably expressing firefly luciferase, mice were treated with or without the TGF-β receptor inhibitor SB431542 at 6.5 mg/kg daily. Tumor growth and liver metastasis were assessed after 5 weeks of treatment (Fig. 7*A*). *In vivo* imaging showed that mice injected with RalGAPβ-deficient cells exhibited strong luminescence signals at the right and left upper abdomen, presumably at the site of the liver and spleen, respectively. These signals were less evident in mice injected with control cells, indicating the high metastatic capacity of RalGAPβ-deficient cells. Notably, SB431542 treatment markedly reduced the luminescence signals in mice-bearing RalGAPβ-deficient cells (Fig. 7*B*). Autopsy tissue samples showed that SB431542 treatment significantly reduced the spleen and liver weights and liver surface metastatic foci in mice-harboring RalGAPβ-deficient cells (Fig. 7, *C* and *D*). In addition, histological analysis showed that SB431542 treatment significantly reduced the liver metastatic area (Fig. 7*E*). These *in vivo* data suggest that TGF-β1 signaling plays an essential role in RalGAPβ deficiency-stimulated metastasis of PDAC cells.

## Discussion

Many studies have demonstrated that Ral is involved in cancer malignancy, especially in invasion and metastasis (24, 25, 26, 27). It has also been reported that Ral is highly activated in PDAC tissues (18). We have recently demonstrated that PDAC cells with high Ral activity, which were generated by RalGAPβ deficiency, exhibited increased activity of local proliferation and metastasis to the liver and peritoneum (9). However, it has been unclear which downstream pathways of Ral were responsible for the enhanced tumor invasion and metastasis. To address this issue, we here performed an array analysis and found that TGF-β1 production was increased downstream of activated Ral. We showed that the TGF-β1 mRNA levels and TGF-β1 protein levels in culture media were elevated in RalGAPβ-deficient PDAC cells with high Ral activity, compared to controls. Conversely, TGF-β1 mRNA and protein were decreased in RalGAPα2-overexpressing PDAC cells with low Ral activity. Thus, TGF-β1 production depends on Ral activity in PDAC cells.

TGF-β1 is a cytokine that exerts crucial effects on cancer progression (19). In normal and premalignant epithelial cells, TGF-β1 can suppress cell growth and tumorigenesis through multiple mechanisms including cell cycle arrest, apoptosis induction, and activation of autophagy. However, in the late stage of cancer progression, cancer cells become resistant to the cytostatic effects of TGF-β1, and TGF-β1 in turn promotes cancer metastasis by inducing epithelial-mesenchymal transition, angiogenesis, and immune evasion. Thus, the effects of TGF-β1 on cancer development and progression are context-dependent. In the present study, we showed that siRNA-knockdown of TGF-β1 inhibited Ral-enhanced migration and invasion *in vitro*. In addition, pharmacological inhibition of the TGF-β receptor kinase suppressed Ral-enhanced invasion *in vitro* and metastasis *in vivo*, indicating that TGF-β1 produced downstream of Ral plays a positive role in Ral-enhanced PDAC invasion and metastasis.

We have previously shown that the activities of the Rho family small GTPases Rac1 and Cdc42 were elevated in RalGAPβ-deficient PDAC cells (9). These conditions would increase the cell motility and contribute to the enhanced invasion and metastasis of RalGAPβ-deficient PDAC cells. Since Rac1 and Cdc42 are activated downstream of TGF-β1 (28, 29), their activation in RalGAPβ-deficient PDAC cells may be mediated by augmented TGF-β1 signaling.

It has been shown that Ral activation induces JNK phosphorylation in H_2_O_2_- or insulin-stimulated cells (30, 31, 32). On the other hand, Balakireva *et al*. reported that Ral suppressed JNK activation in differentiating sensory organs (33). Moreover, Ral has been reported to inhibit JNK signaling and reduce proinflammatory cytokine production by white adipose tissue (34). Thus, the effects of Ral on JNK phosphorylation depend on the cell type and intracellular environment (35). As shown in the present study, Ral appeared to inhibit JNK phosphorylation in MIA PaCa-2 cells and, to a lesser extent, in PANC-1 cells. In addition, the transcription and secretion of TGF-β1 in these PDAC cells were partially, but significantly, enhanced by the JNK inhibitor SP600125. Thus, Ral-inhibited JNK phosphorylation could contribute to increased TGF-β1 production in PDAC cells. It has been demonstrated that JNK deficiency markedly reduces the binding of c-Jun to the negative regulatory element of the TGF-β1 promoter, thereby increasing TGF-β1 transcription (23, 36). Our results suggest a possible regulatory pathway of Ral that enhances TGF-β1 production through inhibition of JNK phosphorylation. It remains elusive mechanistically how Ral inhibits JNK phosphorylation in PDAC cells.

In summary, we have shown that Ral enhances TGF-β1 production, at least in part, through inhibition of JNK activity, which could contribute to Ral-enhanced PDAC invasion and metastasis. Thus, the Ral to TGF-β1 pathway could play an important role in the progression of PDAC.

## Experimental procedures

### Cell culture and reagents

The human PDAC cell lines MIA PaCa-2 and PANC-1 were obtained from the American Type Culture Collection. We used two lines of RalGAPβ-deficient MIA PaCa-2 cells (KO1 and KO2) and PANC-1 cells (KO1 and KO2) previously established by the CRISPR-Cas9 system (9). Cell lines obtained by the same CRISPR treatment but have no indel mutations in the RalGAPβ genomic region were used as controls. All cells were cultured in Dulbecco’s modified Eagle medium (DMEM; Fujifilm Wako Pure Chemical) containing 10% (v/v) fetal bovine serum (FBS; Gibco), 100 U/ml penicillin, and 100 μg/ml streptomycin in a humidified atmosphere with 5% CO_2_ at 37 °C. In some experiments, RalGAPβ-WT, RalGAPα2-WT, and RalGAPα2-N1742K mutant (RalGAPα2-MUT) lacking GAP activity were stably expressed in MIA PaCa-2 and PANC-1 cells using lentivirus expression system as described (14). The type I TGF-β receptor inhibitor SB431542 (Cayman Chemical) and the JNK inhibitor SP600125 (Cayman Chemical) were dissolved in dimethyl sulfoxide and used at indicated concentrations. Unless otherwise specified, all other chemicals were purchased from Sigma-Aldrich or Fujifilm Wako Pure Chemical. All experiments with DNA recombination in this study were approved by the Committees of Tohoku University.

### Microarray analysis

Total RNAs were extracted using Isospin Tissue & Cell RNA extraction kit (Nippon Gene). Clariom S human array (Thermo Fisher Scientific) was used for the microarray analysis. Probe labeling, chip hybridization, and scanning were performed according to the manufacturer’s instructions. The array data were generated using the Transcriptome analysis console software (Thermo Fisher Scientific, https://www.thermofisher.cn/cn/zh/home/life-science/microarray-analysis/microarray-analysis-instruments-software-services/microarray-analysis-software/affymetrix-transcriptome-analysis-console-software.html) to analyze gene expression differences (Supporting information).

### Western blot

Western blot was carried out as described (14). Protein concentration was determined by the Bradford’s method (Bio-Rad) using bovine serum albumin as a standard. Antibodies used in the study were listed in Supporting information (Table S4). ImageJ software (https://imagej.nih.gov/ij/index.html) was used to quantify the band intensity of Western blots.

### siRNA transfection

Cells were transfected with three different siRNAs targeting TGFB1 (Silencer Select Predesigned siRNA s14054 as si-TGF-β1-1, s14055 as si-TGF-β1-2, and s14056 as si-TGF-β1-3, Thermo Fisher Scientific) at 10 nM using Lipofectamine RNAiMAX Transfection Reagent (Thermo Fisher Scientific) according to the manufacturer’s instructions. Forty-eight hours after transfection, cells were used for experiments.

### GST-Sec5 pull-down assay

The glutathione-S-transferase (GST)-Sec5 pull-down assay was performed as described (8, 14). Briefly, cultured cells were serum-starved for 6 h and then stimulated with DMEM containing 10% FBS for 5 min at 37 °C. Cells were lysed in buffer containing 0.5% (w/v) Triton X-100, and cell lysates containing 200 μg proteins were incubated for 1 h at 4 °C with glutathione beads coated with 50 μg GST-tagged Ral-binding domain of Sec5. After washing, bead-associated RalA and RalB were analyzed by Western blot to quantify their GTP-bound forms. These experiments were conducted at least three times independently.

### Wound healing assay

Wound healing assays were performed as described (9). Cells at 100% confluence were scratched with a pipette tip, and photomicrographs were taken immediately at a time point of 18 h or 24 h. The widths of the “wound” were measured using ImageJ software. The proportions of the closed scratch areas were calculated as (widths at the beginning－widths after 18 h or 24 h)/widths at the beginning × 100%. Each experiment was conducted in triplicate and performed three times independently.

### Transwell invasion assay

Transwell invasion assays were performed as described (9). Cells were serum-starved for 6 h and suspended in serum-free DMEM. The cell suspension (MIA PaCa-2: 2 × 10^5^ cells, PANC-1: 5 × 10^4^ cells) was then added to the insert of a transwell chamber (pore size 8 μm, Corning) and incubated for 24 h with DMEM containing 10% FBS in the bottom of the chamber. Cells invading through the Matrigel were fixed with 4% formaldehyde and stained with 0.1% crystal violet, and the number of cells in five preselected fields was counted under a microscope. Each experiment was conducted in triplicate and performed three times independently.

### Quantitative real-time PCR

Total RNA was extracted and reverse transcription was performed using ReverTra Ace qPCR RT Master Mix (TOYOBO). qPCR was then carried out in triplicate with TB Green Premix Extaq II kit (Takara) using a StepOne real-time PCR system (Applied Biosystem). GAPDH was used as an endogenous control. Specific primers for qPCR are listed in Supporting information (Table S5). All results were analyzed using the 2^-△△CT^ method, and each experiment was carried out at least three times independently.

### Enzyme-linked immunosorbent assay

Cells were pretreated with or without 10 μM JNK inhibitor SP600125 for 24 h and then cultured in serum-free DMEM for 72 h. The supernatants were collected to measure TGF-β1 by ELISA following the manufacturer’s instructions (RayBiotech).

### Experimental liver metastasis model

All animal experiments were approved by the Committees of Tohoku University. For liver metastasis model of nude mice, a total of 25-week-old BALB/c male nude mice (Japan SLC) were used and divided into four groups after 1 week acclimation. As described (9), 2 × 10^6^ RalGAPβ-KO2 and control MIA PaCa-2 cells stably expressing firefly luciferase were injected into the spleen of the nude mice. After 1 week, nude mice were treated with or without intraperitoneal injection of TGF-β receptor inhibitor SB431542 (6.5 mg/kg) according to the administration schedule shown in Figure 6*A*. *In vivo* imaging was performed every week as described (14). After 5 weeks of administration, mice were sacrificed, and the weights of the liver and spleen and the visible metastatic lesions on each liver surface were measured. Each liver was randomly dissected into twelve parts, fixed in 10% formalin, and embedded in paraffin. The area of metastasis was quantified on the tissues stained with H&E using ImageJ software.

### Statistical analysis

SPSS 26.0 (https://www.ibm.com/cn-zh/spss) was used for analyzing all data. Results were presented as means ± SD. One-way ANOVA with post hoc Dunnett’s multiple comparison test or Tukey’s multiple comparison test was performed for comparisons between groups. *P* ＜ 0.05 was considered to be statistically significant.

## Data availability

Raw data of the microarray analysis were deposited at NCBI gene expression omnibus under accession number GSE223382.

## Supporting information

This article contains supporting information.

## Conflict of interest

The authors declare that they have no conflicts of interest with the contents of this article.

## Supporting information

Supporting information

Supporting information results
