# Supplementary material for: Ral GTPase promotes metastasis of pancreatic ductal adenocarcinoma via elevation of TGF-β1 production
Source: J Biol Chem. 2023 Apr 26;299(6):104754. doi: 10.1016/j.jbc.2023.104754 (PMC10220265; doi:10.1016/j.jbc.2023.104754)
Supplement: Supporting information results [file mmc2.docx]

**Ral GTPase promotes metastasis of pancreatic ductal adenocarcinoma via elevation of TGF-β1 production**

Mingxin Cao^1, 2, 3, 4^, Xinming Li^5^, Duc-Anh Trinh^1^, Shingo Yoshimachi^1, 6^, Kota Goto^1^, Natsumi Sakata^1^, Masaharu Ishida^6^, Hideo Ohtsuka^6^, Michiaki Unno^6^, Yuxia Wang^5^, Ryutaro Shirakawa^1*^, Hisanori Horiuchi^1, 2*^

Supporting information: Supplementary Figure 1-4 and Table 1-5 with legends

**Fig. S1**


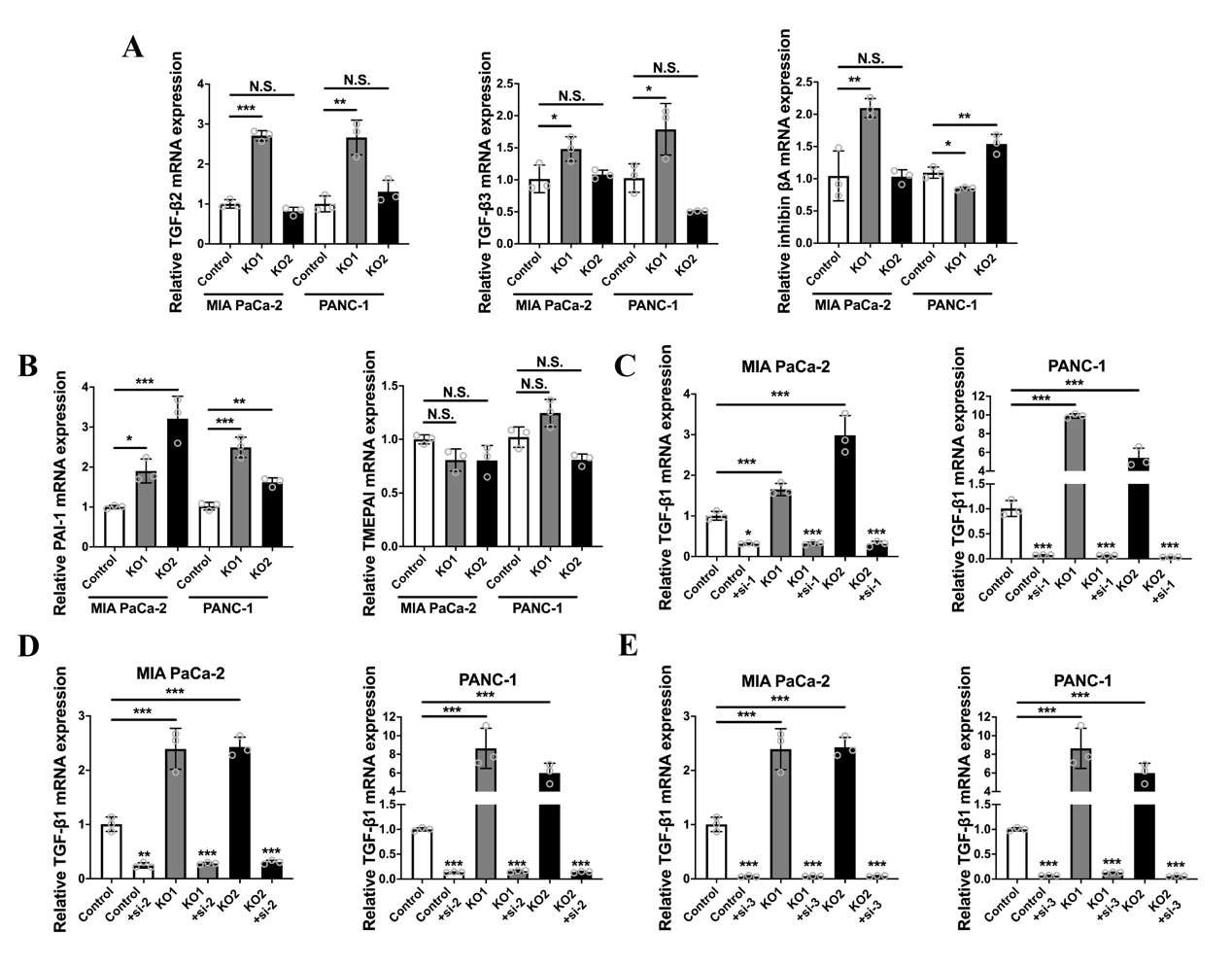


**Supplementary Figure 1. The mRNA levels of typical** **TGF-β family members, target genes, and TGF-β1 after siRNAs knockdown in RalGAPβ-deficient PDAC cells.** mRNA levels of (*A*) TGF-β2, TGF-β3 and inhibin βA, and (*B*) PAI-1 and TMEPAI were quantified in RalGAPβ-deficient and control MIA PaCa-2 and PANC-1 cells by qPCR. *C-E*, TGF-β1 mRNA levels were evaluated by qPCR in RalGAPβ-deficient and control MIA PaCa-2 and PANC-1 cells with or without TGF-β1-siRNA knockdown. Three different siRNAs targeting TGFB1 were used. Comparisons within each cell lines were conducted between control and siRNA-treated group. Data are means ± SD of three independent experiments. N.S., not significant; *, *P*＜0.05; **, *P*＜0.01; ***, *P*＜0.001.

**Fig. S2**

**
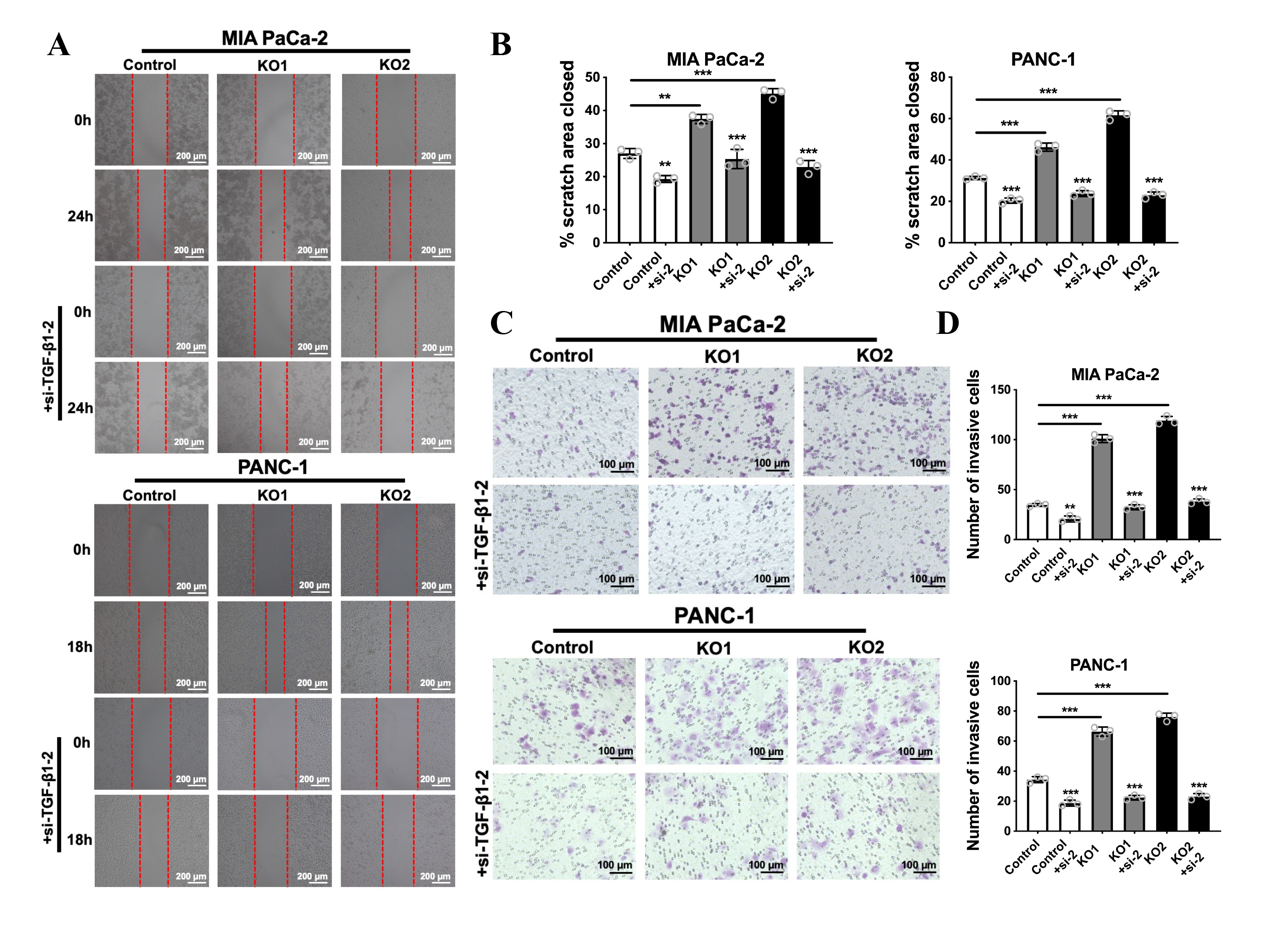
**

**Supplementary Figure 2. Suppression of TGF-β1 expression using siRNA (s14055) attenuates RalGAPβ deficiency-enhanced migration and invasion of PDAC cells.** *A*, Wound healing assay of RalGAPβ-deficient and control MIA PaCa-2 and PANC-1 cells with or without TGF-β1-siRNA knockdown. *B*, Quantification of the closed scratch area (%) of the wound healing assay. *C*, Transwell invasion assay of RalGAPβ-deficient and control MIA PaCa-2 and PANC-1 cells with or without TGF-β1-siRNA knockdown. *D*, Number of invasive cells in the transwell invasion assay. Comparisons within each cell line were conducted between control and siRNA-treated groups. Data are means ± SD of three independent experiments. **, *P*＜0.01; ***, *P*＜0.001.

**Fig. S3**

**
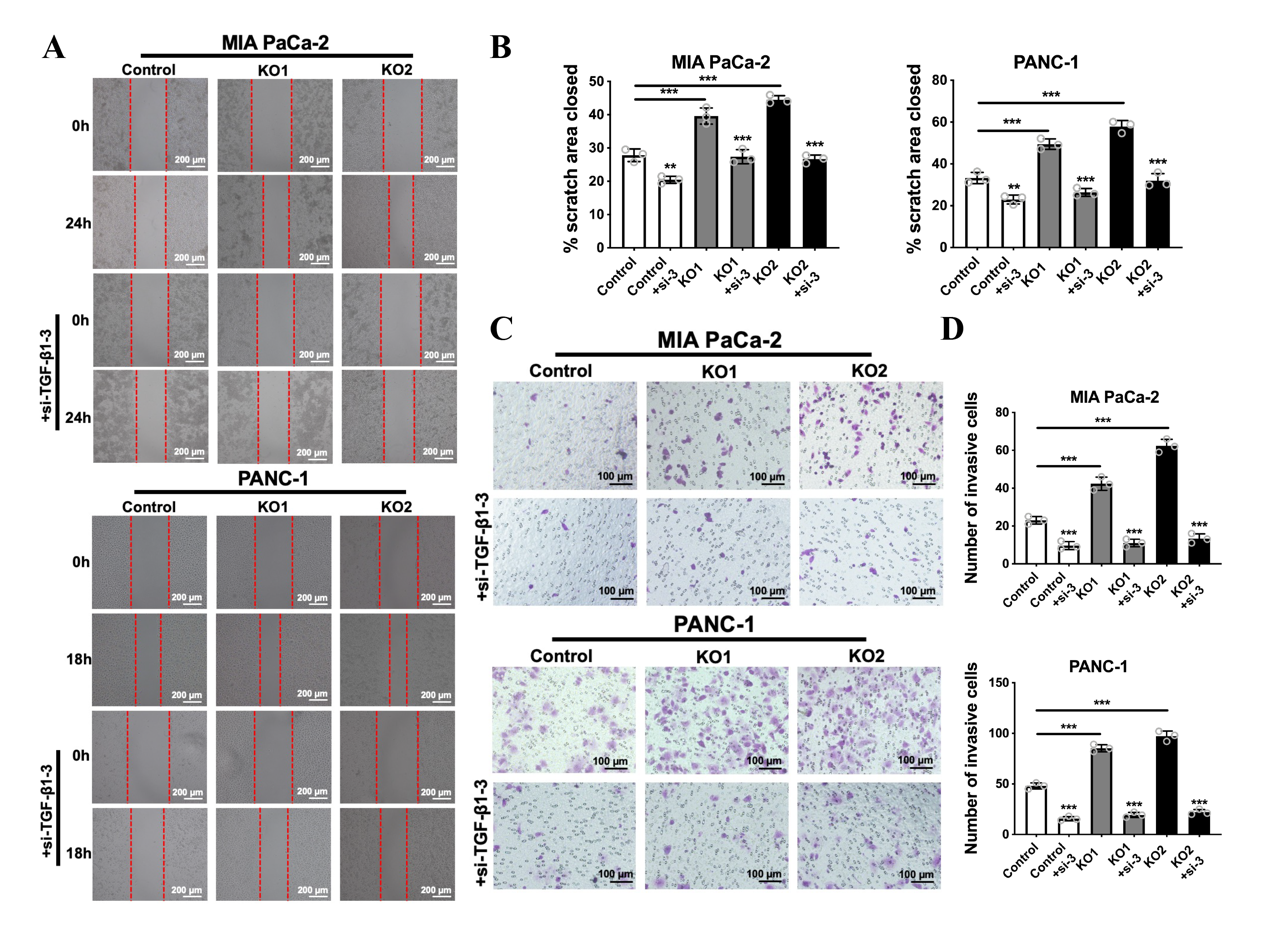
**

**Supplementary Figure 3. Suppression of TGF-β1 expression using siRNA (s14056) attenuates RalGAPβ deficiency-enhanced migration and invasion of PDAC cells.** *A*, Wound healing assay of RalGAPβ-deficient and control MIA PaCa-2 and PANC-1 cells with or without TGF-β1-siRNA knockdown. *B*, Quantification of the closed scratch area (%) of the wound healing assay. *C*, Transwell invasion assay of RalGAPβ-deficient and control MIA PaCa-2 and PANC-1 cells with or without TGF-β1-siRNA knockdown. *D*, Number of invasive cells in the transwell invasion assay. Comparisons within each cell line were conducted between control and siRNA-treated groups. Data are means ± SD of three independent experiments. **, *P*＜0.01; ***, *P*＜0.001.

**Fig. S4**


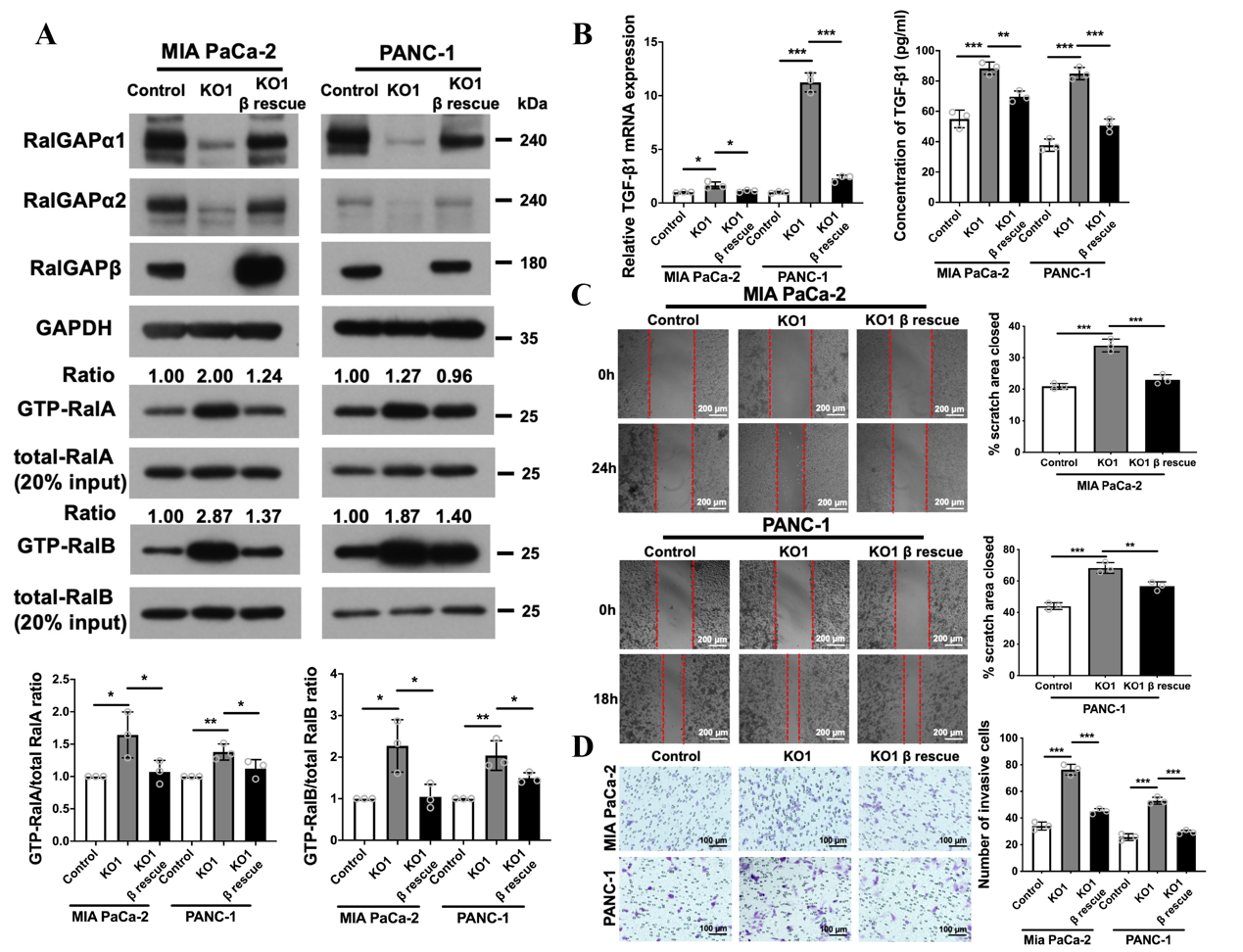


**Supplementary Figure 4. Rescuing RalGAPβ expression in KO1 cells inhibits Ral activation, migration, invasion and TGF-β1 expression.** *A*, GTP-bound RalA and RalB was assessed by GST-Sec5 pull-down assays in control, RalGAPβ-KO1 and RalGAPβ-rescued MIA PaCa-2 and PANC-1 cells. RalGAPα1, RalGAPα2, RalGAPβ, GAPDH, total RalA and RalB were evaluated by Western blot. The ratios of GTP-bound RalA/B to total RalA/B were quantified. *B*, TGF-β1 mRNA and protein levels in the culture media were examined in control, RalGAPβ-KO1 and RalGAPβ-rescued MIA PaCa-2 and PANC-1 cells by qPCR and ELISA. *C* and *D*, Wound healing assay (*C*) and Transwell invasion assay (*D*) of control, RalGAPβ-KO1 and RalGAPβ-rescued MIA PaCa-2 and PANC-1 cells. Representative images are on the left and quantitative results on the right. Data are means ± SD of three independent experiments. *, *P*＜0.05; **, *P*＜0.01; ***, *P*＜0.001.

**Supplementary Table 1. Microarray results of differentially-expressed genes in** **RalGAPβ-deficient cancer cells.**

| **Ratios of quantified mRNA expression** | | **Number of genes** | | | | |
| --- | --- | --- | --- | --- | --- | --- |
|  |  | **MIA PaCa-2 KO1/WT** | **MIA PaCa-2 KO2/WT** | **MIA PaCa-2 KO1/Con** | **MIA PaCa-2 KO2/Con** | **Same results between four groups** |
| Up | ≥ 2.0 | 87 | 211 | 136 | 218 | 8 |
| Down | ≤ -2.0 | 110 | 189 | 84 | 133 | 13 |

(Con = control MIA PaCa-2 cell lines without indel mutation; KO = RalGAPβ-deficient MIA PaCa-2 cell lines by CRISPR-Cas9; WT = parental MIA PaCa-2 cell lines.)

**Supplementary Table 2. Genes that are commonly highly expressed (ratio ≥ 2.0) in RalGAPβ-deficient cancer cells.**

| **Gene name** | **Ratio of quantified mRNA expression** | | | |
| --- | --- | --- | --- | --- |
|  | **MIA PaCa-2 KO1/WT** | **MIA PaCa-2 KO2/WT** | **MIA PaCa-2 KO1/Con** | **MIA PaCa-2 KO2/Con** |
| SLC44A1 | 4.89 | 2.82 | 3.71 | 2.14 |
| FAM129A | 3.54 | 3.16 | 3.42 | 3.06 |
| ELMOD1 | 3.3 | 6.76 | 2.35 | 4.81 |
| CASP4 | 2.71 | 8.45 | 4.53 | 14.14 |
| TGFB1 | 2.55 | 4.12 | 2.66 | 5.69 |
| UBE2L6 | 2.35 | 2.06 | 3.22 | 2.81 |
| GLTP | 2.14 | 2.72 | 2.27 | 2.88 |
| SATB2 | 2.04 | 2.08 | 2.35 | 2.39 |

(Con = control MIA PaCa-2 cell lines without indel mutation; KO = RalGAPβ-deficient MIA PaCa-2 cell lines by CRISPR-Cas9; WT = parental MIA PaCa-2 cell lines.)

**Supplementary Table 3. Genes that are commonly lowly expressed (ratio** **≤ -2.0) in RalGAPβ-deficient cancer cells.**

| **Gene name** | **Ratio of quantified mRNA expression** | | | |
| --- | --- | --- | --- | --- |
|  | **MIA PaCa-2 KO1/WT** | **MIA PaCa-2 KO2/WT** | **MIA PaCa-2 KO1/Con** | **MIA PaCa-2 KO2/Con** |
| C15orf48; MIR147B | -2.13 | -6.36 | -2.44 | -7.28 |
| ACKR3 | -2.2 | -2.53 | -3.29 | -3.79 |
| PPFIBP2 | -2.51 | -2.36 | -5.07 | -4.76 |
| PTPN3 | -2.64 | -6.92 | -2.02 | -5.29 |
| WT1 | -2.66 | -2.41 | -3.03 | -2.74 |
| RBM47 | -2.82 | -4.32 | -2.94 | -4.49 |
| GPER1 | -2.84 | -5.71 | -4.41 | -8.87 |
| ID2 | -2.86 | -3.43 | -3.33 | -3.99 |
| SEMA3D | -3 | -2.74 | -3.03 | -2.77 |
| AP1S3 | -4.36 | -2.31 | -5.43 | -2.87 |
| FZD4 | -6.49 | -2.57 | -7.13 | -2.83 |
| MAP2 | -8.07 | -4.92 | -37.47 | -22.85 |
| SCG2 | -83.34 | -58.21 | -368.08 | -257.08 |

(Con = control MIA PaCa-2 cell lines without indel mutation; KO = RalGAPβ-deficient MIA PaCa-2 cell lines by CRISPR-Cas9; WT = parental MIA PaCa-2 cell lines.)

**Supplementary Table 4. Antibodies used in Western blot**

| **Antibody** | **M.W. (kDa)** | **Source** | **polyclonal/**  **monoclonal** | **Company** | **Catalogue number** |
| --- | --- | --- | --- | --- | --- |
| RalGAPα1 | 240 | Rabbit | polyclonal | Made by ourselves previously (8) | / |
| RalGAPα2 | 220 | Rabbit | polyclonal | Made by ourselves previously (8) | / |
| RalGAPβ | 170 | Rabbit | polyclonal | Made by ourselves previously (8) | / |
| RalA | 28 | Mouse | monoclonal | BD Transduction Laboratories | 610222 |
| RalB | 26 | Rabbit | polyclonal | Cell Signaling Technology | 35235 |
| p-Smad2 (Ser465/467)  /3 (Ser423/425) | 52, 60 | Rabbit | monoclonal | Cell Signaling Technology | 8828 |
| Smad2/3 | 52, 60 | Rabbit | monoclonal | Cell Signaling Technology | 8685 |
| p-JNK (Thr183/Tyr185) | 46, 54 | Mouse | monoclonal | Cell Signaling Technology | 9255 |
| JNK | 46, 54 | Rabbit | polyclonal | Cell Signaling Technology | 9252 |
| GAPDH | 38 | Mouse | monoclonal | Wako Pure Chemical Industries | 015-25473 |

(M.W., molecular weight)

**Supplementary Table 5. Specific primers used for qPCR (5’ to 3’)**

| TGF-β1 | Forward | CTCTCCGACCTGCCACAGA |
| --- | --- | --- |
|  | Reverse | AACCTAGATGGGCGCGATCT |
| TGF-β2 | Forward | CAGCACACTCGATATGGACCA |
|  | Reverse | CCTCGGGCTCAGGATAGTCT |
| TGF-β3 | Forward | AACGGTGATGACCCACGTC |
|  | Reverse | CCGACTCGGTGTTTTCCTGG |
| inhibin βA | Forward | CCTCCCAAAGGATGTACCCA |
|  | Reverse | CTCTATCTCCACATACCCGTTCT |
| PAI-1 | Forward | AAAGGAGCCGTGGACCAGC |
|  | Reverse | TTGGTGAGGGCAGAGAGAGGC |
| TMEPAI | Forward | AGAGCACAGTGTCAGGCAAC |
|  | Reverse | GTGCTGCAGGTACGGATAGG |
| GAPDH | Forward | GAAGGTGAAGGTCGGAGTC |
|  | Reverse | GAAGATGGTGATGGGATTTC |
